# Brain metabolic and functional alterations in a liver-specific PTEN knockout mouse model

**DOI:** 10.1371/journal.pone.0204043

**Published:** 2018-09-20

**Authors:** Ishan Patil, Harsh Sancheti, Bangyan L. Stiles, Enrique Cadenas

**Affiliations:** 1 Pharmacology & Pharmaceutical Sciences, School of Pharmacy, University of Southern California, Los Angeles, CA, United States of America; 2 Medtronic Neurovascular, Medtronic Inc., Irvine, CA, United States of America; University of Basque Country, SPAIN

## Abstract

Insulin resistance–as observed in aging, diabetes, obesity, and other pathophysiological situations, affects brain function, for insulin signaling is responsible for neuronal glucose transport and control of energy homeostasis and is involved in the regulation of neuronal growth and synaptic plasticity. This study investigates brain metabolism and function in a liver-specific Phosphatase and Tensin Homologue (*Pten*) knockout mouse model (Liver-PtenKO), a negative regulator of insulin signaling. The Liver-PtenKO mouse model showed an increased flux of glucose into the liver–thus resulting in an overall hypoglycemic and hypoinsulinemic state–and significantly lower hepatic production of the ketone body beta-hydroxybutyrate (as compared with age-matched control mice). The Liver-PtenKO mice exhibited increased brain glucose uptake, improved rate of glycolysis and flux of metabolites in the TCA cycle, and improved synaptic plasticity in the hippocampus. Brain slices from both control- and Liver-PtenKO mice responded to the addition of insulin (in terms of pAKT/AKT levels), thereby neglecting an insulin resistance scenario. This study underscores the significance of insulin signaling in brain bioenergetics and function and helps recognize deficits in diseases associated with insulin resistance.

## Introduction

The human brain consumes about 25% of the body’s resting state glucose. Neuronal brain glucose uptake occurs mostly through the insulin-sensitive glucose transporter GLUT4 [1]. Stimulation of insulin receptors by insulin leads to activation of the insulin signaling pathway. The subsequent AKT-dependent phosphorylation of several targets results in translocation of GLUT4 from the intracellular storage compartment to the plasma membrane [2]. Once inside the cell, glucose is metabolized via glycolysis and in the tricarboxylic acid cycle, in which the intermediate oxoglutarate can be converted into neurotransmitters, such as glutamate and GABA, thus rendering synaptic plasticity susceptible to the bioenergetic state of the brain [3].

The sensitivity of the brain towards insulin determines its ability to meet the bioenergetic and functional demands of neurons. Insulin has been shown to influence synaptic transmission by modulating the cell membrane expression of NMDA (N-methyl-D-aspartic acid) receptors and affecting long-term potentiation (LTP). Several clinical studies have shown decreased brain glucose uptake to be a common condition in patients with neurodegenerative diseases, such as Alzheimer’s disease (AD) and mild cognitive impairment (MCI) [4,5]. Peripheral insulin resistance is also a typical feature of aging, with several studies suggesting that high circulating insulin and insulin resistance to be important contributors to progressive cognitive impairment and neurodegeneration. Hence, preserving insulin sensitivity is considered as a therapeutic approach for slowing the processes inherent in neurodegeneration [6].

Phosphatase and Tensin Homologue (PTEN) is a negative regulator of the phosphatidylinositol 3-kinase (PI3K)/Akt pathway upon catalyzing the conversion of PI(3,4,5)P_3_ to PI(4,5)P_2_. Deletion of PTEN is known to enhance the activity of the insulin signaling pathway and improve glucose uptake in muscle [7] and adipose cells [8]. A liver-specific deletion of *Pten* in mice also resulted an enhanced liver insulin action, in addition to increased fatty acid synthesis, accompanied by hepatomegaly and a fatty liver phenotype [9,10]. Interestingly, the Liver-PtenKO mouse model also showed lower systemic insulin levels, lower fasting glucose levels, and increased glucose uptake rates in comparison to control mice [11]. Additionally, PTEN loss also improved bioenergetics in hepatocytes [12,13]. Because of their low overall systemic insulin and glucose levels and a more robust insulin signaling, it may be surmised that the brain of the Liver-PtenKO mice would be highly sensitive to insulin.

This study is a proof-of-concept showing that improving insulin signaling and bioenergetics can improve neuronal function, at variance with results from previous studies that described the negative impact on neuronal function of a high-fat diet-induced insulin resistance [14,15]. This study investigates the effect of the unique peripheral phenotype of the liver-specific PTEN knockout mouse model (Liver-PtenKO) on brain metabolism (assessed by ^13^C NMR) and neuronal function (assessed by electrophysiology measurements of long-term potentiation (LTP)). The results underscore the significance of insulin signaling activity and enhanced bioenergetics on synaptic function.

## Materials and methods

### Materials

[1-^13^C]glucose (99%) was purchased from Sigma-Aldrich (St Louis, MO, USA); [1,2-^13^C]acetate (99%) and D_2_O (99.9%) from Cambridge Isotope Laboratories (Andover, MA, USA); the rodent tail vein catheter and restraining apparatus from Braintree Scientific, Inc. (MO, USA); the constant infusion of [1-^13^C]glucose and [1,2-^13^C]acetate was carried out by using a pump from Bio-Rad Laboratories Inc. (CA, USA). All other chemicals were the purest grade available from Sigma-Aldrich.

### Animals

*Pten*^*loxP/loxP*^ mice were bred with *Alb-Cre*^*+/-*^ mice to generate mice with a liver specific deletion [16] and maintained at the University of Southern California (Los Angeles, CA) following NIH guidelines on use of laboratory animals and an approved protocol by the University of Southern California Institutional Animal Care and Use Committee. Mice were housed on 12-h light/dark cycles and provided *ad libitum* access to food and water. 4.5 Month-old mice were used for the experiments. *Pten*^*loxP/loxP*^*; Alb-Cre*^*-*^ were used as control mice. C57BL/6J strain (Jackson Laboratories) mice were used as the background strain to breed the both groups of mice. *Pten*^*loxP/loxP*^*; Alb-Cre*^*+/-*^ mice will be referred to as Liver-PtenKO and the *Pten*^*loxP/loxP*^*; Alb-Cre*^*-*^ as Control (CTL) henceforth.

### Glucose tolerance test (GTT) and ketone body levels

The GTT was performed on the mice after a fasting period of 16 h as previously described [17,18]. For glucose measurement, tail veins were punctured and a small amount of blood was released and applied onto OneTouch glucometer. For the GTT, the mice were given a single dose (2 g/kg of body weight) of D-Dextrose (Sigma Chemical Co.) by i.p. injection after a baseline glucose check. Circulating glucose levels were then measured at 15, 30, 60, and 120 min after glucose injection. Ketone body (beta-hydroxybutyrate) levels were assessed using a colorimetric assay kit (Cayman Chem, 700190).

### Brain glucose uptake

Brain glucose uptake was measured by positron emission tomography utilizing the radiotracer fluoro-2-deoxy-2-[^18^F]-fluoro-D-glucose (FDG-PET) [19] using the Siemens MicroPET R4 PET scanner). After the completion of the FDG-PET scan, the animals underwent CT scanning in the Siemens Inveon microCT scanner, providing information on brain structure and anatomical data. Standard Uptake Values (SUV)–calculated by drawing the regions of interest [20]–represent the standardized uptake value after taking into consideration the actual radioactivity concentration found in the brain at a specific time and the concentration of radioactivity, assuming an even distribution of the injected radioactivity across the whole body.

### Intravenous glucose and acetate infusion and tissue collection and extraction procedure

Infusions were administered as previously described [21,22] on awake and non-anesthetized animals to avoid the effect of anesthesia on cerebral glucose utilization. Animals first received a 0.6 M bolus of [1-^13^C]glucose and [1,2-^13^C]acetate solution to raise the blood glucose levels to normoglycemic range, followed by exponentially decreasing amount of glucose for 8 min. Infusion at a constant rate was performed for 150 min to achieve steady-state concentration of labeled metabolites; at the end of the 150-min infusion, final blood glucose levels were measured. The mouse brain was immediately frozen in liquid nitrogen, and stored at –85°C. Brain tissues were weighed, pulverized to a fine powder, followed by the HClO_4_ extraction procedure. The precipitate was removed by centrifugation (22,000 *g*) and the supernatant was then neutralized to pH 7.4 using KOH. Samples were centrifuged (22,000 *g*) again to separate the supernatant, which formed the final brain extract that was used for NMR analysis.

### NMR spectroscopy and metabolic ratios

The stored brain extracts were thawed and mixed in appropriate proportion with D_2_O, NaN_3_ (preservative), and 1,4-dioxane (chemical shift reference and internal standard). ^13^C NMR analysis was carried out on a Varian VNMRS 600 MHz. ^13^C spectra were acquired with proton-decoupling and nuclear Overhauser enhancement as detailed previously [22]. MestRenova software (Mestrelab Research (CA, USA) was used to integrate relevant peaks after normalization of peak areas. Quantification of each peak was carried out as follows: ^13^C spectra of glutamate, glutamine, aspartate, N-acetyl acetate, lactate, and GABA at natural abundance of ^13^C were acquired in a single solution at different concentrations to construct a standard curve of peak area *versus* ^13^C concentration for each carbon of the compound. The standard curve was used to convert the observed peak area (after normalization using the peak area of 1, 4-dioxane as internal standard) to ^13^C concentration.

The cycling ratio for ^13^C from [1-^13^C] glucose was calculated as follows: ([3-^13^C]glutamate (glutamine)–[1,2-^13^C]glutamate (glutamine))/[4-^13^C]glutamate. The cycling ratio for ^13^C from [1,2-^13^C]acetate was calculated as follows: [1,2-^13^C]glutamate (glutamine)/[4,5-^13^C]glutamate (glutamine). The acetate versus glucose ratios are expressed as [4,5-^13^C]glutamate (glutamine)/[4-^13^C] glutamate (glutamine) and [1,2-^13^C]GABA/ [2-^13^C]GABA [23]. Glycolytic activity was calculated by calculating the % change in levels of [3-^13^C]alanine. The labeling patterns of brain metabolites from [1-^13^C]glucose and [1,2-^13^C]acetate, and their interpretation was carried out as described [24].

### High performance liquid chromatography (HPLC)

HPLC analysis to obtain total (^12^C+^13^C) Glu, Gln, GABA, and Asp concentrations in the brain was measured as previously described [21,25]. Metabolites were quantified by comparison of the peak areas with those of standards. These total metabolite concentrations were used to calculate percentage ^13^C enrichment for ^13^C-labelled metabolite concentrations obtained NMR analysis.

### Long-term potentiation (LTP) and input/output (I/O) curves

LTP assay was performed as previously described [14]: each hippocampal slice was incubated in the incubation chamber for at least 1 h before transferring it to the recording chamber and submerged in a thin layer of ACSF. Field EPSPs (fEPSPs) were recorded from stratum radiatum of CA1 in response to orthodromic stimulation of the Schaffer collateral-commissural projections. I/O curves were generated using stimulus intensities from 100–350 μA in increments of 50 μA using pulses of 0.1 ms duration every 20 s. LTP was induced at baseline intensity using Theta Burst Stimulation (TBS) consisting of ten trains of five 100 Hz stimulation repeated at 5 Hz. Post-stimulation recordings continued for at least 30 min following TBS. fEPSP slope magnitude was calculated as the difference between two cursors, separated by 1 ms, and placed on the middle portion of the ascending phase of the fEPSP. LTP was expressed as a percentage of the average slope after TBS to that of baseline recordings. Groups were compared using repeated measures ANOVA (across all post-theta burst time points). Post-TBS recordings final 5 min were used to calculate the average change between groups for the average change in fEPSP amplitudes. This analysis was followed by a post-hoc t-test for statistical significance.

### Insulin sensitivity assay

400 μm-thick hippocampal regions brain slices–obtained using a vibratome (Series 1000, St Louis, MO)–were exposed to 10 nM insulin in artificial cerebrospinal fluid ACSF for 10 min at room temperature, followed by flash freezing in liquid nitrogen for protein extraction later. Frozen brain tissues were pulverized in liquid nitrogen to a fine powder consistency; ~40 mg of pulverized tissue was collected in 2.0 ml Eppendorf tubes and 200 μl of T-PER (Thermo Fisher Scientific) (containing protease and phosphatase inhibitor cocktails (Sigma Aldrich) in a 1:100 ratio) was added. Tubes were allowed to sit on ice for 10 min, followed by centrifugation at 10,000*g* for 10 min at 4°C. Supernatant protein concentration was measured by a BCA protein assay (Thermo Scientific, IL). Quantitative measure of phosphorylated and total AKT was carried out by the Phospho(Ser^473^)/Total AKT multiplex assay kit (Meso Scale Discovery^®^, K15100D).

### Data analysis

GraphPad Prism version 7.0 was used to analyze data. Student's two-tailed t-test was used for statistical analysis of paired data. The level of statistical significance and the values of *n* are indicated in the respective figure (*p ≤ 0.05, **p ≤ 0.01).

## Results

### Glucose tolerance test and ketone body levels

**Fig 1:**
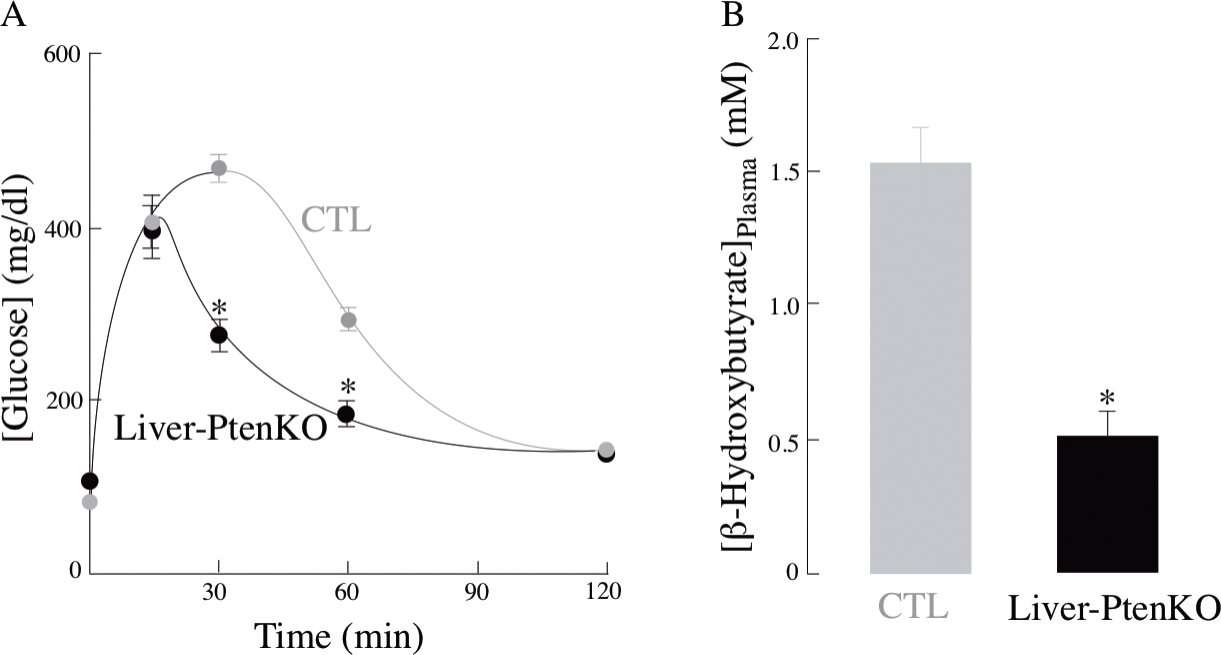
Faster plasma glucose clearance and fewer ketone body plasma levels in the Liver-PtenKO mouse model. (A) Plasma glucose clearance was faster in the Liver-PtenKO mice and (B) plasma ketone body levels were significantly lower than in the Liver-PtenKO mice. Statistical significance calculated using student t-test (*P ≤ 0.05; *n* = 5 per group). Error bars indicate ± SD.

The LiverPten-KO mouse model has been extensively characterized [11,26]. There were not statistically significant differences in body weight between control and LiverPten-KO mice. A glucose tolerance test was performed to assess the differences in ability of these mice to absorb glucose from blood over time; glucose clearance from blood proceeded faster with the Liver-PtenKO mice as compared with the CTL mice (Fig 1), in agreement with previous reports [11,26]. The Liver-PtenKO mice showed significantly faster rate of glucose absorption at the 30 min and 60 min mark, with glucose clearance rates normalizing to those seen in the CTL mice around the 120-min mark. These changes can be attributed to a more robust insulin signaling activity in the Liver-PtenKO livers due to *Pten* deletion, resulting in higher glucose uptake by liver. Plasma ketone body concentrations were significantly lower in the Liver-PtenKO than in the CTL mice (Fig 1) as expected from a robust glucose metabolism in liver.

### Brain glucose uptake

**Fig 2:**
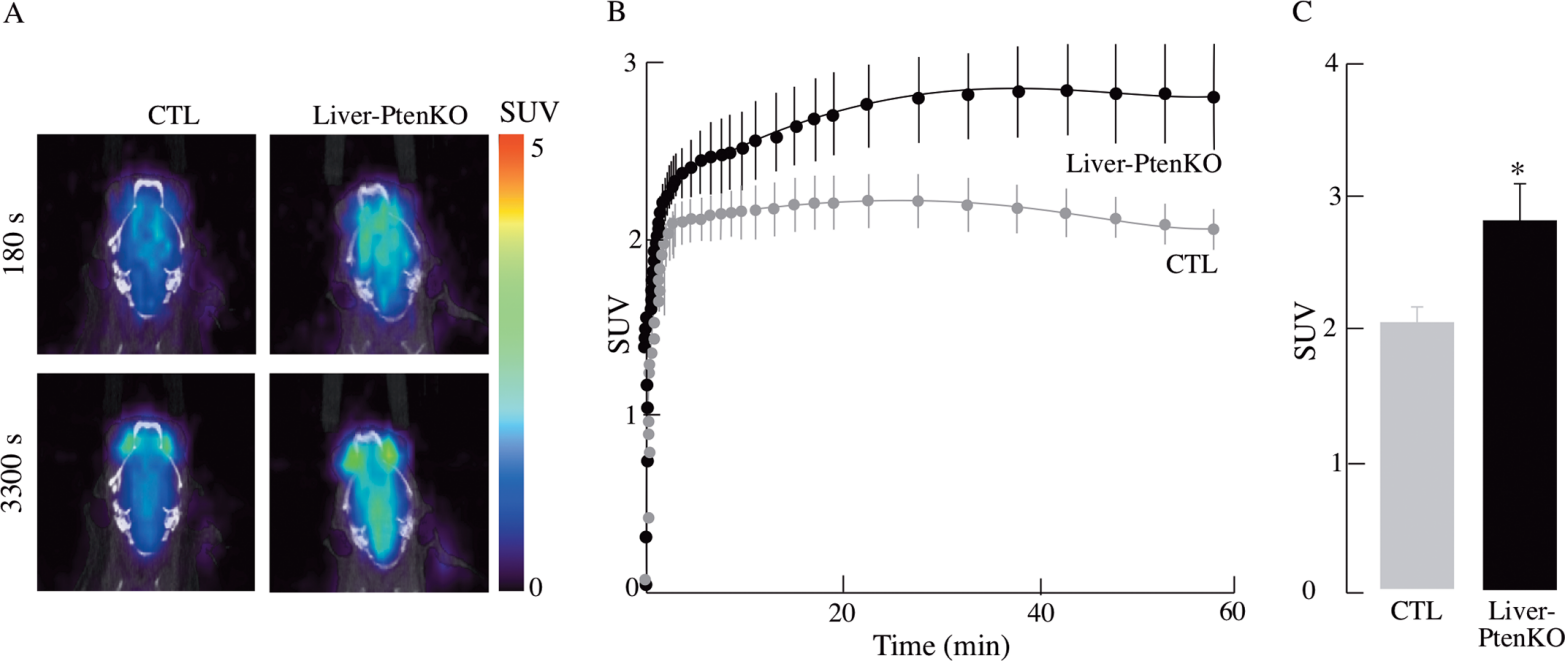
Dynamic [^18^F]-FDG-PET imaging of CTL and Liver-PtenKO mouse brains. Standard uptake value (SUV) was calculated after [^18^F]-FDG injection followed by dynamic PET and CT scanning. (A) Representative combined images from PET-CT scanning of CTL and Liver-PtenKO at 180 and 3300 s into [^18^F]- PET scan. (B) Average rates of uptake of glucose by brains of CTL and Liver-PtenKO mice reveal significantly faster rates of glucose uptake in the Liver-PtenKO brains, with error bars indicating ± SEM. (C) Total glucose uptake at the end of Dynamic [^18^F]-FDG scan duration show Liver-PtenKO brains taking up glucose in larger quantities than CTL mice brains (*n* = 4 per group). Error bars indicate ± SD.

Dynamic [^18^F]-FDG-PET imaging was used to assess the rate of brain glucose uptake in Liver-PtenKO- and CTL mice (*n* = 4 for each group). Liver-PtenKO showed a higher rate of brain glucose uptake (Fig 2) and significantly greater total glucose absorption, represented as standard uptake values (SUV). These results suggest a higher glucose/insulin-sensitive brain in the Liver-PtenKO mouse model.

### ^13^C Labelling of brain metabolites

^13^C-NMR spectroscopy was used assess the differences in brain energy metabolism between the two groups. Both groups of mice were infused with a solution [1-^13^C]glucose and [1,2-^13^C]acetate for 150 min, after which the brains were extracted to isolate metabolites. The 150 min time point was chosen as the end point to allow for sufficient enrichment of all ^13^C-metabolites and to help examine all possible differences in the metabolic characteristics between *Liver-PtenKO*- and CTL mice [21]. ^13^C labeled isotopomers of lactate, glutamate (Glu), glutamine (Gln), aspartate (Asp), gamma-aminobutyric acid (GABA), N-acetyl-aspartate (NAA), myoinositol (MI), and glucose (C1alpha and beta) were observed. Table 1 shows the concentrations (expressed in mM ± SEM) of different ^13^C-labelled isotopomers of Glu, Gln, Asp, NAA, GABA and MI in the 4.5 month-old Liver-PtenKO- and CTL mice. Greater overall labeling was observed in the Liver-PtenKO samples than in the age-matched CTL mice.

**Table 1:** Concentrations of different isotopomers of ^13^C Glu, Gln, Asp, NAA, GABA, and MI in mouse brains after 150-min infusion of [1-^13^C]glucose and [1,2-^13^C]acetate quantified by ^13^C-NMR spectroscopy.

Metabolite	CTL	Liver-PtenKO	CTL vs Liver-PtenKO	% change
[4-^13^C]Glu	1.489 ± 0.17	1.940 ± 0.09	0.007 (*)	30.289
[3-^13^C]Glu	1.147 ± 0.07	1.381 ± 0.05	0.009 (*)	20.401
[2-^13^C]Glu	1.128 ± 0.09	1.384 ± 0.012	0.042 (*)	22.695
[1-^13^C]Glu	0.630 ± 0.04	0.717 ± 0.01	0.010 (*)	13.810
[4,5-^13^C]Glu	0.427 ± 0.04	0.517 ± 0.03	0.118	21.077
[2,3-^13^C]Glu	0.525 ± 0.08	0.839 ± 0.07	0.003 (*)	59.810
[3,4-^13^C]Glu	0.784 ± 0.13	1.073 ± 0.105	0.028 (*)	36.862
[1,2-^13^C]Glu	0.378 ± 0.06	0.507 ± 0.08	0.065	34.127
[4-^13^C]Gln	0.506 ± 0.07	0.550 ± 0.008	0.307	8.696
[3-^13^C]Gln	0.521 ± 0.05	0.594 ± 0.01	0.057	14.012
[2-^13^C]Gln	0.503 ± 0.02	0.597 ± 0.04	0.010 (*)	18.688
[1-^13^C]Gln	0.338 ±0.04	0.384 ± 0.02	0.116	13.609
[4,5-^13^C]Gln	0.339 ± 0.07	0.380 ± 0.06	0.382	12.094
[2,3-^13^C]Gln	0.220 ± 0.04	0.278 ± 0.03	0.077	36.364
[3,4-^13^C]Gln	0.210 ± 0.25	0.445 ± 0.008	0.166	111.905
[1,2-^13^C]Gln	0.222 ± 0.05	0.258 ± 0.07	0.430	16.216
[4-^13^C]Asp	0.296 ± 0.07	0.298 ± 0.03	0.960	0.676
[3-^13^C]Asp	0.403 ± 0.03	0.433 ± 0.02	0.212	7.444
[2-^13^C]Asp	0.283 ± 0.02	0.315 ± 0.01	0.058	11.307
[1-^13^C]Asp	0.281 ± 0.09	0.236 ± 0.04	0.470	-16.014
[3,4-^13^C]Asp	0.110 ± 0.03	0.113 ± 0.04	0.879	2.727
[2,3-^13^C]Asp	0.157 ± 0.03	0.204 ± 0.06	0.527	29.936
[1,2-^13^C]Asp	0.064 ± 0.02	0.111 ± 0.002	0.020 (*)	73.438
[3-^13^C]NAA	0.123 ± 0.02	0.132 ± 0.06	0.764	7.317
[2-^13^C]NAA	0.082 ± 0.02	0.089 ± 0.001	0.614	11.307
[6-^13^C]NAA	0.115 ± 0.02	0.111 ± 0.02	0.813	-3.478
[4-^13^C]GABA	0.280 ± 0.03	0.342 ± 0.08	0.192	22.143
[3-^13^C]GABA	0.347 ± 0.02	0.370 ± 0.06	0.470	6.628
[2-^13^C]GABA	0.337 ± 0.04	0.380 ± 0.03	0.209	12.760
[1-^13^C]GABA	0.209 ± 0.03	0.342 ± 0.08	0.821	63.636
[3,4-^13^C]GABA	0.280 ± 0.001	0.040 ± 0.02	0.195	-85.714
[2,3-^13^C]GABA	0.132 ± 0.008	0.118 ± 0.04	0.522	-10.606
[1,2-^13^C]GABA	0.099 ± 0.04	0.095 ± 0.08	0.931	-4.040
[4,6-^13^C]MI	0.097 ± 0.006	0.117 ± 0.02	0.098	20.619
[2-^13^C]MI	0.040 ± 0.01	0.035 ± 0.001	0.567	-12.500
[1,3-^13^C]MI	0.105 ± 0.02	0.117 ± 0.02	0.493	11.429
[5-^13^C]MI	0.056 ± 0.02	0.070 ± 0.001	0.416	25.000

ASP, aspartate; GABA, gamma-aminobutyric acid; Gln, glutamine; Glu, glutamate; MI, myoino-sitol; NAA, N-acetylaspaartate. Daga in columns 2 and 3 are presented as average mM ± SD. Column 4 data are *P* values obtained from a two-tailed student t test after comparing between the CTL and Liver-PtenKO groups.

*P ≤ 0.05. *n* = 4 per group. Error bars indicate ± SD.

**Fig 3:**
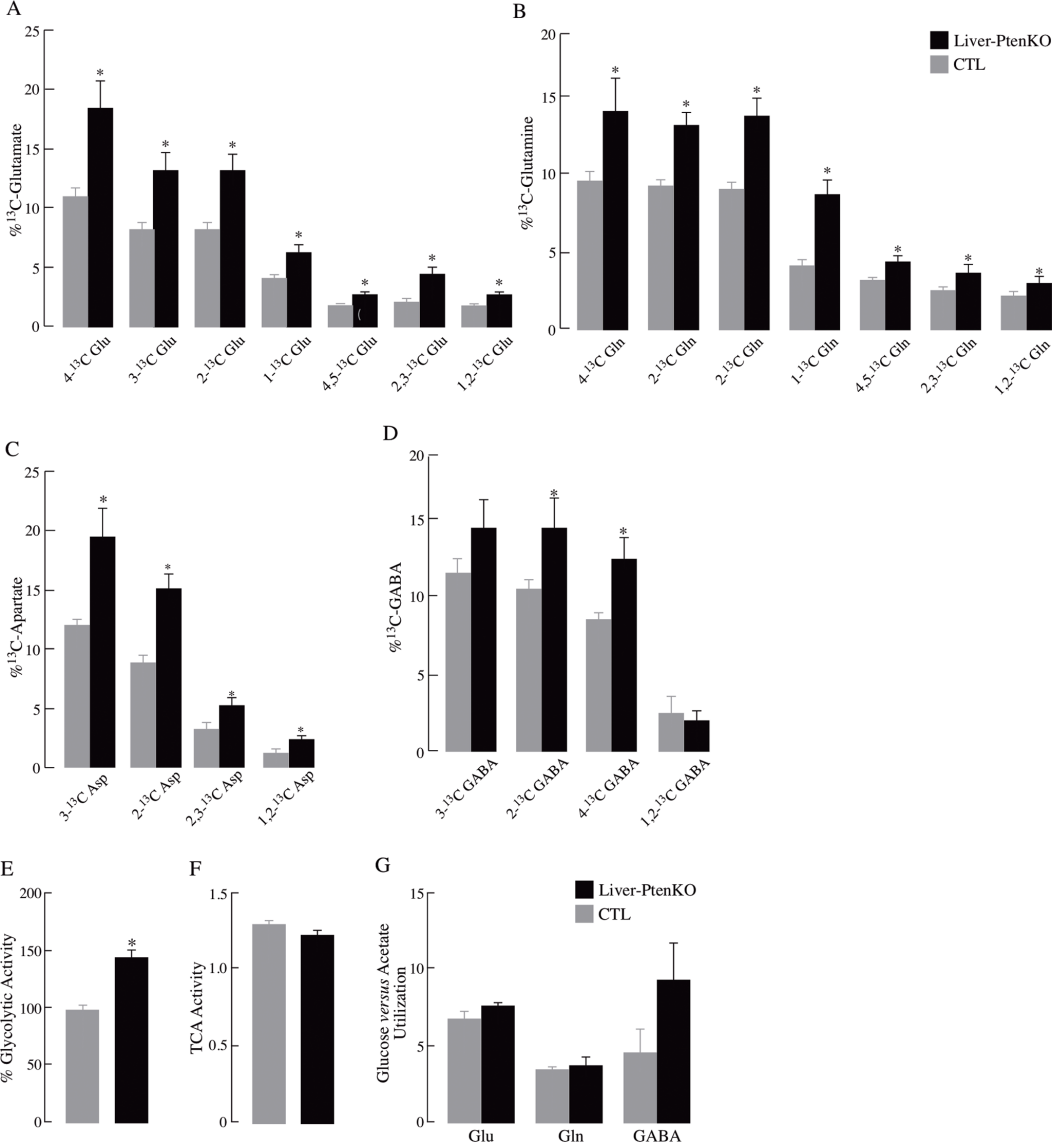
Increased percentage enrichment of ^13^C labeled isotopomers in Liver-PtenKO mouse brains. Overall, an increase in the enrichment of 13-C labeled isotopomers of (A) Glutamate, (B) Glutamine, (C) Aspartate, and (D) GABA after [1-^13^C] glucose + [1,2-^13^C] acetate infusion observed the brains of the Liver-PtenKO mice suggests a possible hypermetabolic state in the brain. Metabolic ratios, calculated as described in the materials and methods section, after [1-^13^C]glucose + [1,2-^13^C]acetate infusion for 150 min are shown in the Graphs E-G. (E) % Glycolytic activity based on the levels of [3-^13^C]alanine. (F) TCA cycle activity. (G) Glucose versus acetate utilization for formation of Glu, Gln and GABA. Statistical significance calculated using paired student t-test (*P ≤ 0.05; *n* = 4 per group). Error bars indicate ± SD.

The enrichments of these metabolites reflect the relative content of different labeled isotopomers as a fraction of the total concentration of metabolites. The total concentrations [^12^C + ^13^C] of glutamate, glutamine, GABA, and aspartate (Table 2) were analyzed by HPLC, and no significant differences were found in the total metabolite concentrations between the two groups. The fractional enrichment (%) of the various ^13^C-labelled metabolite isotopomers of glutamate (Fig 3A), glutamine (Fig 3B), aspartate (Fig 3C), and GABA (Fig 3D) were quantified based on the absolute concentrations. The fractional enrichment values reveal the actual differences in the metabolic states of neurons and astrocytes. The brains of the Liver-PtenKO mice showed significantly higher levels of [4-^13^C]-Glu, [3-^13^C]-Glu, [2-^13^C]-Glu, [1-^13^C]-Glu, [4,5-^13^C]-Glu, [2,3-^13^C]-Glu, [1,2-^13^C]-Glu, [4-^13^C]-Gln, [3-^13^C]-Gln, [2-^13^C]-Gln, [1-^13^C]-Gln, [4,5-^13^C]-Gln, [3-^13^C]-Asp, [2-^13^C]-Asp, [2,3-^13^C]-Asp, [1,2-^13^C]-Asp, [2-^13^C]-GABA, [4-^13^C]-GABA.

**Table 2:** Total concentrations of [^12^C + ^13^C] of glutamate, glutamine, GABA, and aspartate; n = 4 per group.

Metabolite	CTL	Liver-PtenKO	P value (*≤0.05)
Aspartate	2.72 ± 0.54	2.23 ± 0.29	0.11
Glutamate	12.21 ± 1.55	10.39 ± 1.44	0.14
Glutamine	4.99 ± 0.20	4.38 ± 0.65	0.08
GABA	2.74 ± 0.16	2.48 ± 0.39	0.22

A comparison between neuronal and glial metabolism can also be assessed using the co-infusion of [1-^13^C]glucose and [1,2-^13^C]acetate. A possible overall hypermetabolic state was observed in both neurons and astrocytes of the Liver-PtenKO in comparison to the CTL brains, as seen by the significant increase in the levels of the ^13^C-labelled metabolite isotopomers at the end of the 150-min infusion (Table 1; Fig 3).

Glucose is taken up by both the neurons and astrocytes [27]. The majority of acetyl Coenzyme A (acetyl CoA) is metabolized from glucose in neurons [28]. [1-^13^C]glucose ultimately gives rise to [4-^13^C]glutamate, which can be converted to [2-^13^C]GABA in GABAergic neurons. In the Liver-PtenKO brains, all single-labeled glutamate isotopomers labeled in the 1^st^, 2^nd^, and 3^rd^ turns showed greater concentrations of ^13^C-labelling by an average of 25%, in comparison to those in the CTL mice. Levels of [2-^13^C]GABA were much higher in the Liver-PtenKO brains as well, albeit not statistically significant. The higher concentrations of these labeled metabolites indicate the presence of a faster neuronal metabolism in the Liver-PtenKO mouse model.

[1,2-^13^C]acetate is predominantly taken up and metabolized by astrocytes and is ultimately converted to [4,5-^13^C]glutamate and [4,5-^13^C]glutamine [29,30]. [4,5-^13^C]glutamine transferred to neurons is converted back to [4,5-^13^C]glutamate for use as a neurotransmitter and further to [1,2-^13^C]GABA in GABAergic neurons. The levels of [4,5-^13^C] glutamine and [2,3-^13^C] glutamate reflect metabolites originating from astrocytic metabolism and were found to be significantly higher in concentration in the Liver-PtenKO mice. In addition, the concentration of [2-^13^C]glutamine, metabolized from [1-^13^C]glucose in astrocytes, was greater in the Liver-PtenKO brains. These changes reveal that astrocytes play a more prominent role in supporting neuronal metabolism in the Liver-PtenKO brains in comparison to the astrocytes in the CTL brains as well as a likely hypermetabolic state in the brain of the Liver-PtenKO mice.

### Metabolic ratios

Metabolic ratios were calculated based on the concentration of different isotopomers after infusion ^13^C-labeled glucose and acetate. Liver-PtenKO mice showed higher glycolytic activity (calculated as the % change in the concentration of [3-^13^C]alanine) (Fig 3E). The cycling ratio provides information of how long the label stays in the TCA cycle before getting converted to glutamate or glutamine but there were no significant differences between CTL and Liver-PtenKO (Fig 3F). The acetate versus glucose utilization ratio provides an estimate of the relative contribution from neurons and astrocytes to glutamate, glutamine and GABA formation. A slight increase was observed in the ^13^C cycling ratios for both glucose and acetate metabolism and in the glucose *versus* acetate utilization index for Glu, Gln, and GABA but not statistically significant (Fig 3G).

### Long-term potentiation (LTP) and hippocampal synaptic plasticity

**Fig 4:**
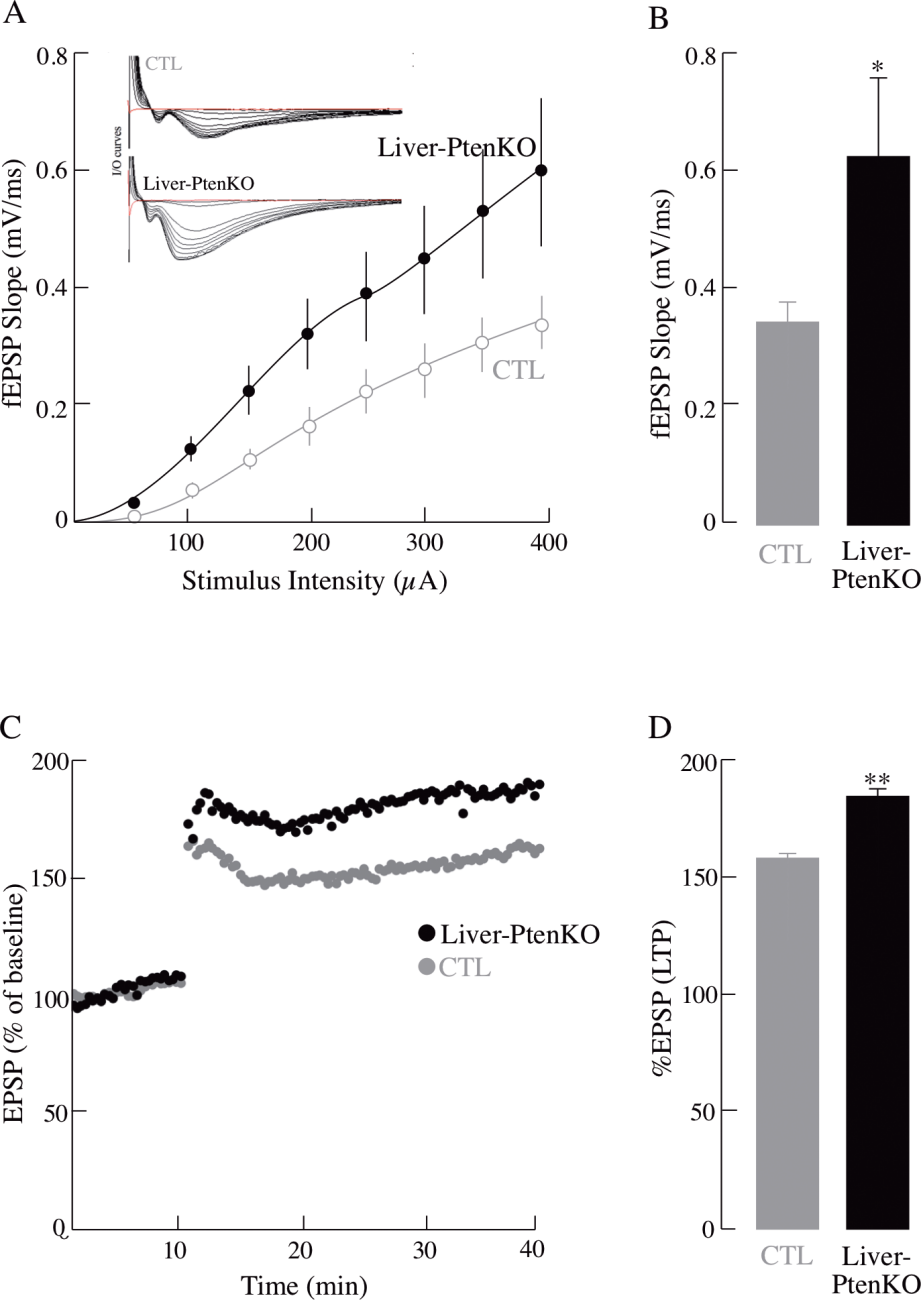
Improved hippocampal synaptic plasticity in Liver-PtenKO mouse model. Input/Output (I/O) and LTP changes were measured in both groups. (A) Representative I/O curves for both groups at increasing stimulus intensities, and I/O slope values at increasing stimulus intensities. (B) Average I/O curve slopes for both groups at 400 μA. (C) LTP induced at baseline intensity using theta burst stimulation (TBS) consisting of ten trains of five 100 Hz stimulation repeated at 5 Hz. Slope of EPSPs was measured and results normalized to the average value measured during the 10 min baseline period. (D) Average of the last 5 min of recordings post-TBS, which is considered as LTP. Total *n* = 20 slices; *n* = 10 slices/group and at *n* = 4 animals/group. Statistical significance calculated using paired student t-test (*P ≤ 0.05; **P ≤ 0.01). Error bars indicate ± SD.

The LTP assay was used to assess the difference in the physiological outcome (synaptic plasticity) because of the different peripheral phenotypes of the two groups. Insulin signaling plays an important role in modulating brain synaptic plasticity, which can be assessed by changes in long-term-potentiation (LTP). Synaptic plasticity can be measured using electrophysiological experiments by examining Input/Output (I/O) responses at baseline and after theta burst-stimulation (TBS) in the hippocampal CA1 region. The ability of neurons to maintain a high output response after a high frequency electrical stimulation for a prolonged length of time is indicative of strength of synaptic transmission and plasticity. The Liver-PtenKO were able to produce a significantly higher I/O response and a steeper fEPSP slope in comparison to the CTL mice (Fig 4A and 4B). In addition, the Liver-PtenKO manifested a substantially higher LTP value than that of the CTL mice represented as %fEPSP values (Fig 4C and 4D). These data show that a liver-specific deletion of *Pten* in the Liver-PtenKO, resulted in hippocampal neurons developing better synaptic plasticity than that of the CTL mice.

### Brain insulin sensitivity

**Fig 5:**
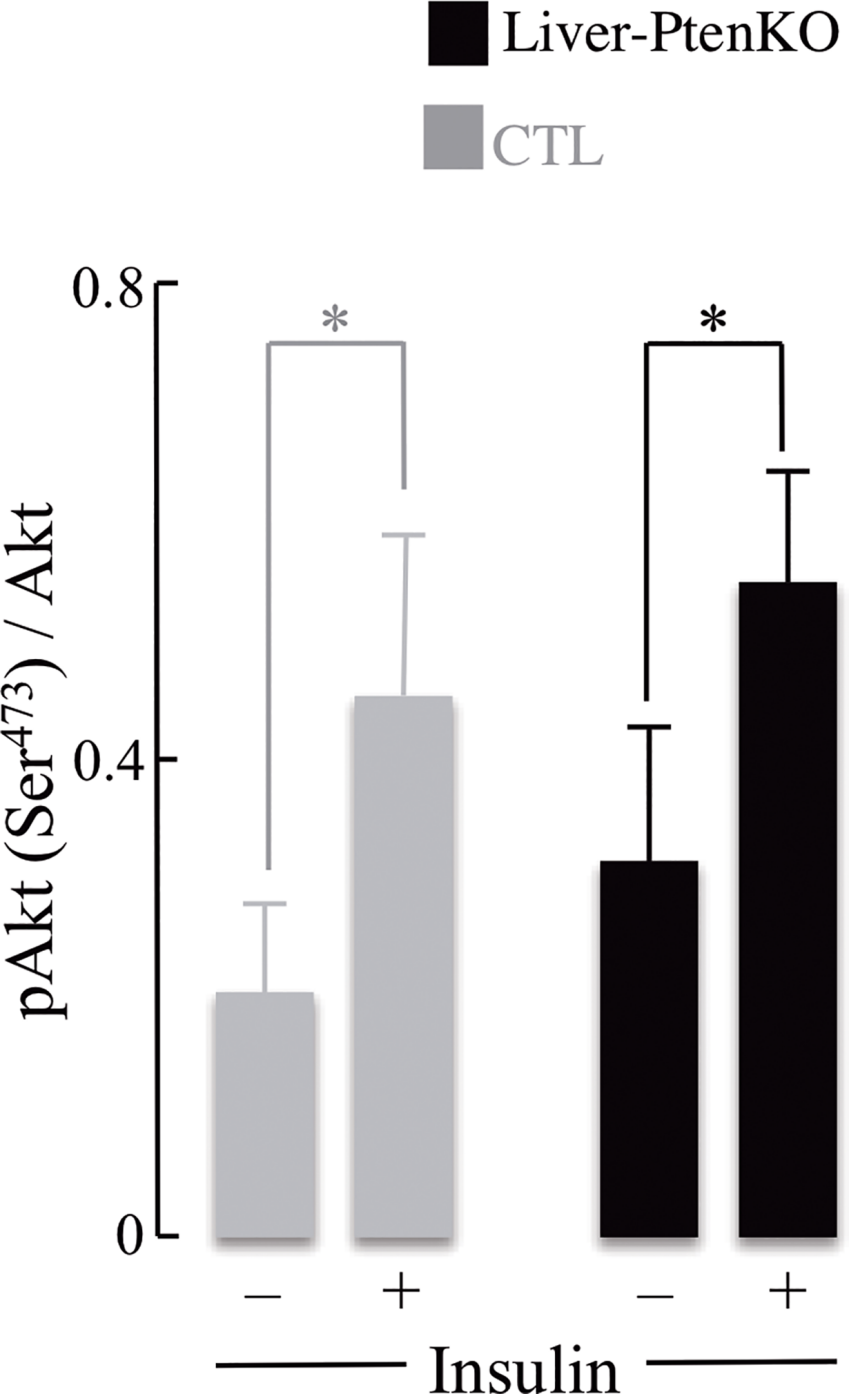
Insulin stimulation of brain slices from CTL and Liver-PtenKO mice. Levels of p-AKT (Ser^473^) and total AKT were measured using a plate assay in whole brain protein extracts with or without 10 nM insulin treatment. pAKT/AKT ratios indicate significant changes after insulin treatment within groups, but no significant differences in ratios between CTL (gray) and Liver-PtenKO (black) groups. Error bars indicate ± SD (*P ≤ 0.05) (*n* = 4 per group).

The brain insulin sensitivity assay was used to assess changes in brain PI3K-AKT signaling activity and insulin responsiveness. Fig 5 depicts changes in the ratios of p-AKT(Ser^473^)/AKT in both groups, with and without insulin stimulation. An increase in AKT phosphorylation (response to insulin supplementation of brain slices) was observed in both CTL and the Liver-PtenKO brains; hence, the ratios of p-AKT/AKT, with or without insulin stimulation, were similar in both groups. These results indicate that insulin signaling activity in the Liver-PtenKO brains (baseline levels) is slightly more robust than in the CTL mice, but not more sensitive to insulin, for the p-AKT/AKT ratios remained similar to those in the CTL mice after insulin stimulation in both groups. Comparison between groups (CTL and Liver-PtenKO) was not statistically significant.

## Discussion

A liver specific deletion of *Pten* resulted in improved liver insulin signaling activity leading to higher glucose absorption in the liver, and increased glycogen synthesis, whereas the levels triglyceride, leptin, insulin, and fasting glucose levels to be decreased in the plasma of the *Pten*^*loxP/loxP*^*;Alb-Cre*^*+*^ mutant mice. The *Pten* deletion has been shown to be specific to the liver, with no leakage to other tissues, including the brain [11,31,32]. This study was aimed at establishing the effect of this unique peripheral phenotype on brain metabolism and synaptic plasticity. The Liver-PtenKO model in this study entails a relatively hypoglycemic and hypo-insulinemic state that may force the brain to become more insulin- or glucose-sensitive, hence, it may be surmised that the Liver-PtenKO phenotype will directly improve synaptic plasticity and function in the mutant mice. We have previously reported that impairments in brain metabolism, due to an induced insulin resistant state, can negatively affect synaptic plasticity [14,15].

The notion of an insulin- or glucose-sensitive brain in the Liver-PtenKO mice stems from (*a*) the dynamic [^18^F]-FDG-PET imaging (Fig 2), where brain glucose uptake in the Liver-PtenKO mice proceeded at a faster rate than that of the CTL mice; (*b*) the prominent increase in the enrichment of ^13^C isotopomers of Glu and Gln showing that more label was transferred to these metabolites in the Liver-PtenKO mice as compared to the CTL mice (Table 1, Fig 3), thereby suggesting that the Liver-PtenKO mice were more proficient in incorporating and metabolizing glucose and acetate for energetic purposes. Both, neuronal and the supporting astrocytic metabolism was faster in the Liver-PtenKO mice. Although not statistically significant, neuronal glycolytic activity was also higher in the Liver-PtenKO mice; (*c*) brain insulin sensitivity, measured by calculating the pAKT(Ser^473^)/AKT ratio (Fig 5), revealed the absence of insulin resistance and the occurrence of a more robust insulin signaling activity in the Liver-PtenKO mice, but not of enhanced responsiveness to insulin stimulation, as seen by similar ratios of p-AKT/AKT expression with or without insulin stimulation. This is at variance with a high-fat diet mouse model, in which the addition of insulin to brain slices failed to elicit an increase the pAKT/AKT values [15]; (*d*) functional outcome consequential to the increase brain glucose uptake, enhanced neuronal and astrocytic metabolism, and lack of brain insulin resistance, is manifested in the electrophysiological studies (Fig 4) that revealed better I/O responses from the hippocampal CA1 neurons in the Liver-PtenKO mice and significantly higher LTP. We [14] and others [33] have reported a decrease in LTP following peripheral insulin resistance in high-fat diet (HFD) models. A co-infusion of [1-^13^C]glucose and [1,2-^13^C]acetate and NMR-spectroscopy quantification in HFD-fed mouse brains from a previous study [15] revealed the development of an overall hypermetabolic state, but exhibited reduced brain glucose uptake, indicative of brain insulin resistance. Then the only difference in the functional outcome (LTP) between the Liver-PtenKO mice and the high-fat diet-fed mice can be attributed to the absence of central and brain insulin resistance and the occurrence of a robust insulin signaling pathway. Hence, the data in this study emphasize the importance of the insulin signaling pathway and the prominent role it plays in modulating neuronal function and synaptic plasticity.

It is important to acknowledge that the Liver-PtenKO mice develop a fatty liver phenotype with age [11]. However, the fatty liver phenotype observed in the Liver-PtenKO is different (*i*.*e*. hypoinsulinemia, enhanced insulin signaling, and low plasma non-esterified fatty acid levels or NEFAs) from that observed in the models of naturally occurring fatty liver states (*i*.*e*., hyperinsulinemic and insulin-resistant). Mechanistically, hepatic insulin resistance is known to dysregulate lipid metabolism, leading to lipolysis [34], which then leads to increased production of toxic lipids such as ceramides that have been reported to impair insulin signaling and mitochondrial function [35–38]. The fatty liver development in the Liver-PtenKO mice is due to an enhanced insulin signal (resulting for PTEN loss) in hepatocytes, leading to increased de novo synthesis of lipids mediated by AKT2 [26,31,39]. Steatophepatitis and hepatic insulin resistance can be caused by various etiologies including hepatitis C infection, alcohol abuse, obesity, and nitrosamine exposures, which are associated with cognitive impairment and neuropsychiatric disorders [40–43]. In fact, cognitive impairment and neuropsychiatric disorders correlate more with steatohepatitis and insulin resistance rather than with T2DM or obesity [44,45]. Of significance for this study, the Liver-PtenKO mice displays systemic insulin sensitive rather than resistance phenotype due to the enhanced insulin/PI3K signal in the liver, indicating that all the effects observed in the brain are a result of the peripheral phenotype of the Liver-PtenKO mice.

With increasing incidence rates of neurodegenerative diseases like AD and insulin resistance disease states such as type 2 diabetes, obesity, non-alcoholic fatty liver disease, and metabolic syndrome, this study underscores the importance of preserving insulin sensitivity and the integrity of the insulin signaling pathway [46]. Clinical studies show decreased brain glucose uptake to be a common condition in patients with Alzheimer’s disease and mild cognitive impairment [4,5]. In fact, multiple studies have indicated metabolic dysfunction and bioenergetic deficits as antecedents to development of Alzheimer’s pathology and related dementias [47–50]. Because insulin resistance can affect cognition and brain function through disruption of brain glucose uptake and metabolism, this study definitely fuels the concept of treating brain insulin resistance as a therapeutic approach for slowing the process inherent in neurodegeneration [6].

## Abbreviations

aCSF: artificial cerebrospinal fluid
Asp: aspartate
EPSP: excitatory postsynaptic potential
fEPSP: field EPSPs
GABA: gamma-aminobutyric acid
Glu: glutamate
Gln: glutamine
GLUT: glucose transporter
HFD: high-fat diet
I/O: input/output
LTP: long-term potentiation
NMR: nuclear magnetic resonance
NAA: N-acetyl aspartate
Pet/CT: positron emission tomography/computed tomography.

## References

[1] Bingham EM, Hopkins D, Smith D, Pernet A, Hallett W, Reed L, et al The role of insulin in human brain glucose metabolism: an 18fluoro-deoxyglucose positron emission tomography study. Diabetes. 2002;51:3384–90. . 12453890 10.2337/diabetes.51.12.3384

[2] Rowland AF, Fazakerley DJ, James DE. Mapping insulin/GLUT4 circuitry. Traffic. 2011;12:672–81. 10.1111/j.1600-0854.2011.01178.x . 21401839

[3] Schubert D. Glucose metabolism and Alzheimer's disease. Ageing Res Rev. 2005;4:240–57. 10.1016/j.arr.2005.02.003 15950548

[4] Mosconi L, Mistur R, Switalski R, Tsui WH, Glodzik L, Li Y, et al FDG-PET changes in brain glucose metabolism from normal cognition to pathologically verified Alzheimer's disease. Eur J Nucl Med Mol Imaging. 2009;36:811–22. 10.1007/s00259-008-1039-z ; PMCID: PMC2774795. 19142633 PMC2774795

[5] Mosconi L. Brain glucose metabolism in the early and specific diagnosis of Alzheimer’s disease. Eur J Nucl Med Mol Imaging. 2005;32(4):486–510. 10.1007/s00259-005-1762-7 15747152

[6] Stefanelli M, Martocchia A, De Marinis EA, Falaschi GM, Romano G, Rufo M, et al Treatment of insulin resistance in the neurodegeneration. Recent Pat CNS Drug Discov. 2014;9(1):54–63. . 24724584 10.2174/1574889809666140410093006

[7] Wijesekara N, Konrad D, Eweida M, Jefferies C, Liadis N, Giacca A, et al Muscle-specific Pten deletion protects against insulin resistance and diabetes. Mol Cell Biol. 2005;25:1135–45. 10.1128/MCB.25.3.1135-1145.2005 ; PMCID: PMCPMC544010. 15657439 PMC544010

[8] Kurlawalla-Martinez C, Stiles B, Wang Y, Devaskar SU, Kahn BB, Wu H. Insulin hypersensitivity and resistance to streptozotocin-induced diabetes in mice lacking PTEN in adipose tissue. Mol Cell Biol 2005;25:2498–510. 10.1128/MCB.25.6.2498-2510.2005 ; PMCID: PMC1061603. 15743841 PMC1061603

[9] Galicia VA, He L, Dang H, Kanel G, Vendryes C, French BA, et al Expansion of hepatic tumor progenitor cells in Pten-null mice requires liver injury and is reversed by loss of AKT2. Gastroenterology. 2010;139(6):2170–82. 10.1053/j.gastro.2010.09.002 ; PMCID: PMC2997180. 20837017 PMC2997180

[10] Stiles B, Wang Y, Stahl A, Bassilian S, Lee WP, Kim YJ, et al Liver-specific deletion of negative regulator Pten results in fatty liver and insulin hypersensitivity [corrected]. Proc Natl Acad Sci U S A. 2004;101(7):2082–7. 10.1073/pnas.0308617100 ; PMCID: PMCPMC357055. 14769918 PMC357055

[11] Stiles B, Wang Y, Stahl A, Bassilian S, Lee WP, Kim YJ, et al Liver-specific deletion of negative regulator Pten results in fatty liver and insulin hypersensitivity. Proc Natl Acad Sci USA. 2004;101:2082–7. 10.1073/pnas.0308617100 . 14769918 PMC357055

[12] Li C, Li Y, He L, Agarwal AR, Zeng N, Cadenas E, et al PI3K/AKT signaling regulates bioenergetics in immortalized hepatocytes. Free Radic Biol Med. 2013;60:29–40. 10.1016/j.freeradbiomed.2013.01.013 ; PMCID: PMCPMC3654039. 23376468 PMC3654039

[13] Li Y, He L, Zeng N, Sahu D, Cadenas E, Shearn C, et al Phosphatase and tensin homolog deleted on chromosome 10 (PTEN) signaling regulates mitochondrial biogenesis and respiration via estrogen-related receptor alpha (ERRalpha). J Biol Chem. 2013;288(35):25007–24. 10.1074/jbc.M113.450353 ; PMCID: PMCPMC3757167. 23836899 PMC3757167

[14] Liu Z, Patil IY, Jiang T, Sancheti H, Walsh JP, Stiles BL, et al High-fat diet induces hepatic insulin resistance and impairment of synaptic plasticity. PLoS One. 2015;10(5):e0128274 10.1371/journal.pone.0128274 ; PMCID: PMC4449222. 26023930 PMC4449222

[15] Liu Z, Patil I, Sancheti H, Yin F, Cadenas E. Effects of Lipoic Acid on High-Fat Diet-Induced Alteration of Synaptic Plasticity and Brain Glucose Metabolism: A PET/CT and (13)C-NMR Study. Sci Rep. 2017;7(1):5391 10.1038/s41598-017-05217-z ; PMCID: PMCPMC5511189. 28710347 PMC5511189

[16] Debebe A, Medina V, Chen CY, Mahajan IM, Jia C, Fu D, et al Wnt/beta-catenin activation and macrophage induction during liver cancer development following steatosis. Oncogene. 2017;36(43):6020–9. 10.1038/onc.2017.207 ; PMCID: PMCPMC5666317. 28671671 PMC5666317

[17] Zeng N, Li Y, He L, Xu X, Galicia V, Deng C, et al Adaptive basal phosphorylation of eIF2alpha is responsible for resistance to cellular stress-induced cell death in Pten-null hepatocytes. Mol Cancer Res. 2011;9(12):1708–17. 10.1158/1541-7786.MCR-11-0299 ; PMCID: PMCPMC4351767. 22009178 PMC4351767

[18] Yang KT, Bayan JA, Zeng N, Aggarwal R, He L, Peng Z, et al Adult-onset deletion of Pten increases islet mass and beta cell proliferation in mice. Diabetologia. 2014;57(2):352–61. 10.1007/s00125-013-3085-8 ; PMCID: PMCPMC3918745. 24162585 PMC3918745

[19] Jiang T, Yin F, Yao J, Brinton RD, Cadenas E. Lipoic acid restores age-associated impairment of brain energy metabolism through the modulation of Akt/JNK signaling and PGC1a transcriptional pathway. Aging Cell. 2013;12:1021–31. 10.1111/acel.12127 23815272 PMC3819405

[20] Sancheti H, Akopian G, Yin F, Brinton RD, Walsh JP, Cadenas E. Age-dependent modulation of synaptic plasticity and insulin mimetic effect of lipoic acid on a mouse model of Alzheimer's disease. PLoS One. 2013;8(7):e69830 Epub 2013/07/23. 10.1371/journal.pone.0069830 ; PMCID: PMCPMC3714252. 23875003 PMC3714252

[21] Sancheti H, Kanamori K, Patil I, Díaz Brinton R, Ross BD, Cadenas E. Reversal of metabolic deficits by lipoic acid in a triple transgenic mouse model of Alzheimer's disease: a (13)C NMR study. J Cereb Blood Flow Metab. 2014;34(2):288–96. 10.1038/jcbfm.2013.196 PMID: PMC3915206. 24220168 PMC3915206

[22] Sancheti H, Patil I, Kanamori K, Díaz Brinton R, Zhang W, Lin AL, et al Hypermetabolic state in the 7-month-old triple transgenic mouse model of Alzheimer's disease and the effect of lipoic acid: a (13)C-NMR study. J Cereb Blood Flow Metab. 2014;34(11):1749–60. 10.1038/jcbfm.2014.137 ; PMCID: PMCPMC4269751. 25099753 PMC4269751

[23] Kondziella D, Brenner E, Eyjolfsson EM, Markinhuhta KR, Carlsson ML, Sonnewald U. Glial-neuronal interactions are impaired in the schizophrenia model of repeated MK801 exposure. Neuropsychopharmacology. 2006;31(9):1880–7. 10.1038/sj.npp.1300993 . 16395297

[24] Nilsen LH, Rae C, Ittner LM, Götz J, Sonnewald U. Glutamate metabolism is impaired in transgenic mice with tau hyperphosphorylation. J Cereb Blood Flow Metab. 2013;33:684–91. 10.1038/jcbfm.2012.212 23340677 PMC3652703

[25] Kanamori K, Ross BD. The first in vivo observation of ^13^C-^15^N coupling in mammalian brain. J Magn Reson. 2001;153:193–202. 10.1006/jmre.2001.2432 11740894

[26] He L, Hou X, Kanel G, Zeng N, Galicia V, Wang Y, et al The critical role of AKT2 in hepatic steatosis induced by PTEN loss. Am J Pathol. 2010;176(5):2302–8. 10.2353/ajpath.2010.090931 ; PMCID: PMCPMC2861095. 20348245 PMC2861095

[27] Nehlig A, Wittendorp-Rechenmann E, Lam CD. Selective uptake of [14C]2-deoxyglucose by neurons and astrocytes: high-resolution microautoradiographic imaging by cellular 14C-trajectography combined with immunohistochemistry. J Cereb Blood Flow Metab. 2004;24(9):1004–14. Epub 2004/09/10. 10.1097/01.WCB.0000128533.84196.D8 15356421

[28] Qu H, Haberg A, Haraldseth O, Unsgard G, Sonnewald U. (13)C MR spectroscopy study of lactate as substrate for rat brain. Dev Neurosci. 2000;22(5–6):429–36. Epub 2000/12/09. 10.1159/000017472 . 11111159

[29] Hassel B, Bachelard H, Jones P, Fonnum F, Sonnewald U. Trafficking of amino acids between neurons and glia in vivo. Effects of inhibition of glial metabolism by fluoroacetate. J Cereb Blood Flow Metab. 1997;17(11):1230–8. Epub 1997/12/09. 10.1097/00004647-199711000-00012 . 9390655

[30] Waniewski RA, Martin DL. Preferential utilization of acetate by astrocytes is attributable to transport. J Neurosci. 1998;18(14):5225–33. Epub 1998/07/03. . 9651205 10.1523/JNEUROSCI.18-14-05225.1998 PMC6793490

[31] Moon BC, Hernandez-Ono A, Stiles B, Wu H, Ginsberg HN. Apolipoprotein B secretion is regulated by hepatic triglyceride, and not insulin, in a model of increased hepatic insulin signaling. Arterioscler Thromb Vasc Biol. 2012;32(2):236–46. 10.1161/ATVBAHA.111.241356 ; PMCID: PMCPMC3870321. 22155452 PMC3870321

[32] Stiles B, Wang Y, Stahl A, Bassilian S, Lee WP, Kim Y-J, et al Liver-specific deletion of negative regulator Pten results in fatty liver and insulin hypersensitivity. Proc Natl Acad Sci USA. 2004;101(7):2082–7. 10.1073/pnas.0308617100 14769918 PMC357055

[33] Porter DW, Kerr BD, Flatt PR, Holscher C, Gault VA. Four weeks administration of liraglutide improves memory and learning as well as glycaemic control in mice with high fat dietary-induced obesity and insulin resistance. Diabetes Obesity Metab. 2010;12:891–9. 10.1111/j.1463-1326.2010.01259.x 20920042

[34] Kao Y, Youson JH, Holmes JA, Al-Mahrouki A, Sheridan MA. Effects of insulin on lipid metabolism of larvae and metamorphosing landlocked sea lamprey, Petromyzon marinus. Gen Comp Endocrinol. 1999;114(3):405–14. . 10336828 10.1006/gcen.1999.7265

[35] Kraegen EW, Cooney GJ. Free fatty acids and skeletal muscle insulin resistance. Curr Opin Lipidol. 2008;19(3):235–41. 10.1097/01.mol.0000319118.44995.9a . 18460913

[36] Holland WL, Summers SA. Sphingolipids, insulin resistance, and metabolic disease: new insights from in vivo manipulation of sphingolipid metabolism. Endocr Rev. 2008;29(4):381–402. 10.1210/er.2007-0025 ; PMCID: PMC2528849. 18451260 PMC2528849

[37] Langeveld M, Aerts JM. Glycosphingolipids and insulin resistance. Prog Lipid Res. 2009;48:196–205. 10.1016/j.plipres.2009.03.002 . 19303901

[38] de la Monte SM. Triangulated Mal-Signaling in Alzheimer's Disease: Roles of Neurotoxic Ceramides, ER Stress, and Insulin Resistance Reviewed. J Alzheimers Dis. 2012;30(0):S231–S49. 10.3233/jad-2012-111727 22337830 PMC4550324

[39] Palian BM, Rohira AD, Johnson SA, He L, Zheng N, Dubeau L, et al Maf1 is a novel target of PTEN and PI3K signaling that negatively regulates oncogenesis and lipid metabolism. PLoS Genet. 2014;10(12):e1004789 10.1371/journal.pgen.1004789 ; PMCID: PMCPMC4263377. 25502566 PMC4263377

[40] Tong M, Neusner A, Longato L, Lawton M, Wands JR, de la Monte SM. Nitrosamine exposure causes insulin resistance diseases: relevance to type 2 diabetes mellitus, non-alcoholic steatohepatitis, and Alzheimer's disease. J Alzheimers Dis. 2009;17(4):827–44. ; PMCID: PMC2952429. 20387270 PMC2952429

[41] Weiss J, Gorman J. Psychiatric behavioral aspects of comanagement of hepatitis C virus and HIV. Current HIV/AIDS Rep. 2006;3(4):176–81. 10.1007/s11904-006-0013-2 PMC2601635 17032577

[42] Perry W, Hilsabeck R, Hassanein T. Cognitive Dysfunction in Chronic Hepatitis C: A Review. Dig Dis Sci. 2008;53(2):307–21. 10.1007/s10620-007-9896-z 17703362

[43] Tong M, Longato L, de la Monte SM. Early limited nitrosamine exposures exacerbate high fat diet-mediated type 2 diabetes and neurodegeneration. BMC endocrine disorders. 2010;10:4 10.1186/1472-6823-10-4 ; PMCID: PMC3161394. 20302640 PMC3161394

[44] Schmidt KS, Gallo JL, Ferri C, Giovannetti T, Sestito N, Libon DJ, et al The neuropsychological profile of alcohol-related dementia suggests cortical and subcortical pathology. Dement Geriatr Cogn Disord. 2005;20:286–91. 10.1159/000088306 . 16166775

[45] Kopelman MD, Thomson AD, Guerrini I, Marshall EJ. The Korsakoff syndrome: clinical aspects, psychology and treatment. Alcohol. 2009;44(2):148–54. 10.1093/alcalc/agn118 . 19151162

[46] de la Monte SM, Tong M. Brain metabolic dysfunction at the core of Alzheimer's disease. Biochem Pharmacol. 2014;88(4):548–59. 10.1016/j.bcp.2013.12.012 . 24380887 PMC4550323

[47] Galindo MF, Ikuta I, Zhu X, Casadesus G, Jordan J. Mitochondrial biology in Alzheimer's disease pathogenesis. J Neurochem. 2010;114(4):933–45. Epub 2010/05/25. 10.1111/j.1471-4159.2010.06814.x . 20492350

[48] Gibson GE, Sheu K-FR, Blass JP. Abnormalities of mitochondrial enzymes in Alzheimer disease. J Neural Transn. 1998;105(8):855–70. 10.1007/s007020050099 9869323

[49] Hauptmann S, Scherping I, Dröse S, Brandt U, Schulz KL, Jendrach M, et al Mitochondrial dysfunction: An early event in Alzheimer pathology accumulates with age in AD transgenic mice. Neurobiol Aging. 2009;30:1574–86. 10.1016/j.neurobiolaging.2007.12.005 PMCID: PMCPMID . 18295378

[50] Yao J, Irwin RW, Zhao L, Nilsen J, Hamilton RT, Brinton RD. Mitochondrial bioenergetic deficit precedes Alzheimer's pathology in female mouse model of Alzheimer's disease. Proc Natl Acad Sci USA. 2009;106(34):14670–5. 10.1073/pnas.0903563106 ; PMCID: PMCPMC2732886. 19667196 PMC2732886

